# Recombinant Factor VIII Fc Inhibits B Cell Activation via Engagement of the FcγRIIB Receptor

**DOI:** 10.3389/fimmu.2020.00138

**Published:** 2020-02-07

**Authors:** Maria T. Georgescu, Paul C. Moorehead, Tongyao Liu, Jennifer Dumont, David W. Scott, Christine Hough, David Lillicrap

**Affiliations:** ^1^Clinical and Molecular Hemostasis Research Group, Department of Pathology and Molecular Medicine, Queen's University, Kingston, ON, Canada; ^2^Janeway Children's Health and Rehabilitation Centre, St. John's, NL, Canada; ^3^Faculty of Medicine, Memorial University, St. John's, NL, Canada; ^4^Bioverativ, a Sanofi Company, Cambridge, MA, United States; ^5^Department of Medicine, Uniformed Services University, Bethesda, MD, United States

**Keywords:** hemophilia A—complications, drug therapy, anti-drug antibodies, factor VIII inhibitors, recombinant factor VIII Fc, FcγRIIB, B cell inhibition

## Abstract

The development of neutralizing antibodies (inhibitors) against factor VIII (FVIII) is a major complication of hemophilia A treatment. The sole clinical therapy to restore FVIII tolerance in patients with inhibitors remains immune tolerance induction (ITI) which is expensive, difficult to administer and not always successful. Although not fully understood, the mechanism of ITI is thought to rely on inhibition of FVIII-specific B cells (1). Its efficacy might therefore be improved through more aggressive B cell suppression. FcγRIIB is an inhibitory Fc receptor that down-regulates B cell signaling when cross-linked with the B cell receptor (BCR). We sought to investigate if recombinant FVIII Fc (rFVIIIFc), an Fc fusion molecule composed of FVIII and the Fc region of immunoglobulin G1 (IgG1) (2), is able to inhibit B cell activation more readily than FVIII. rFVIIIFc was able to bind FVIII-exposed and naïve B cells from hemophilia A mice as well as a FVIII-specific murine B cell hybridoma line (413 cells). An anti-FcγRIIB antibody and FVIII inhibited binding, suggesting that rFVIIIFc is able to interact with both FcγRIIB and the BCR. Furthermore, incubation of B cells from FVIII-exposed mice and 413 cells with rFVIIIFc resulted in increased phosphorylation of SH-2 containing inositol 5-phosphatase (SHIP) when compared to FVIII. B cells from FVIII-exposed hemophilia A mice also exhibited decreased extracellular signal-regulated kinase (ERK) phosphorylation when exposed to rFVIIIFc. These differences were absent in B cells from naïve, non-FVIII exposed hemophilic mice suggesting an antigen-dependent effect. Finally, rFVIIIFc was able to inhibit B cell calcium flux induced by anti-Ig F(ab)_2_. Our results therefore indicate that rFVIIIFc is able to crosslink FcγRIIB and the BCR of FVIII-specific B cells, causing inhibitory signaling in these cells.

## Introduction

Hemophilia A is an inherited bleeding disorder caused by defects or deficiencies in factor VIII (FVIII), an essential protein co-factor of the intrinsic coagulation pathway. Affected individuals experience prolonged provoked hemorrhages, and in severe cases spontaneous bleeding into joints and soft tissues. Although FVIII replacement can be used to mitigate these symptoms, the development of inhibitory antibodies remains a major complication of this therapy, occurring in 30% of patients with severe disease (3). Bleeding symptoms in this subset of individuals can be treated with bypassing agents such as FVIII inhibitory bypassing activity (FEIBA) (4) or recombinant activated factor VII (rFVIIa) (5), which drive clot formation via the extrinsic coagulation pathway. However, these are very expensive products that offer inferior and inconsistent hemostatic protection compared to FVIII. Restoring tolerance to the protein and thus re-enabling FVIII replacement therapy is the preferred management option for hemophilia A patients with inhibitors.

Immune tolerance induction (ITI) remains the only therapy to desensitize hemophilia A patients who develop an immune response to FVIII. This approach consists of repeated and often daily administration of high [200 IU/kg (6)] or low [50 IU/kg (7)] doses of FVIII. The treatment is continued for prolonged periods of time ranging from weeks to years (8), until the inhibitor is eradicated and the recovery as well as half-life of FVIII normalize. ITI is expensive, difficult to administer, lowers quality of life and can be complicated by events such as central venous catheter infections (9). In addition, this therapy is effective in only 70–85% of cases (10). As a result, methods to increase ITI efficacy would be of great benefit.

Despite its long-term use in clinical practice, the immunological mechanisms underlying ITI are not fully understood. There are data to suggest that successful tolerance induction is associated with the generation of anti-idiotypic antibodies (11, 12) which could neutralize soluble and B cell surface anti-FVIII immunoglobulin (Ig). Studies in murine models of hemophilia A have also shown that high doses of FVIII can inhibit FVIII-specific B cells thereby preventing anti-FVIII IgG production (1). The improved efficacy of ITI when combined with rituximab (anti-CD20 monoclonal antibody) provides further evidence for the importance of B cell eradication in the success of ITI (13). Based on our current understanding of this therapy it is therefore reasonable to conclude that the efficacy of ITI may be increased by improved inhibition or elimination of FVIII-specific B cells.

FcγRIIB is one of the five receptors that can bind the Fc region of IgG and modulate immune responses. Although these receptors are widely expressed by cells of the immune system and have varying functions based on the cell of origin, FcγRIIB is of particular interest as it is the lone inhibitory Fcγ receptor and is the only Fc receptor expressed by B cells (14). When cross-linked with the B cell receptor (BCR) by an antigen-IgG immune complex, FcγRIIB can inhibit B cell activation. This process is mediated by phosphorylation of FcγRIIB's cytoplasmic immunoreceptor tyrosine-based inhibitory motif (ITIM), ultimately resulting in inhibition of proliferation via the MAPK pathway and decreased calcium flux (15). Cross-linking the BCR of FVIII-specific B cells with FcγRIIB might therefore offer an improved potential for inhibiting the activation of these cells. This mechanism could also provide further mechanistic basis for the decreased immunogenicity of rFVIIIFc in pre-clinical models.

Recombinant FVIII Fc (rFVIIIFc) is a fusion protein composed of B domain deleted (BDD) FVIII fused to the Fc region of IgG1. This molecule was designed to increase FVIII half-life through the IgG recycling mechanism mediated by the neonatal Fc receptor (FcRn) (2) in the endosomes of endothelial cells. The addition of IgG1 Fc to FVIII may however also allow this molecule to interact with Fcγ receptors, which could have immunological implications. Preclinical studies have already shown that replacement therapy with rFVIIIFc results in an attenuated immune response when compared to FVIII. This effect was mediated by regulatory T cell, Fcγ receptors, and possibly FcRn (16). Case reports and retrospective studies of hemophilia A patients undergoing ITI with rFVIIIFc have suggested a quicker time to tolerization when compared to ITI using conventional FVIII concentrates (17, 18). Finally, antibodies targeted to FcγRIIB have been shown to modulate the FVIII immune response (19). Based on this evidence we hypothesize that rFVIIIFc may inhibit FVIII-specific B cells more efficiently than FVIII due to its ability to cross-link the BCR of these cells with FcγRIIB.

## Methods

### Animals

Hemophilia A mice with an exon 16 knockout of the *F8* gene on a C57Bl6 background were used for all experiments (20). FVIII-exposed mice were generated by administering 6 IU/dose (~200 IU/kg) of FVIII (Advate, Takeda) IV for 4 consecutive weeks (21). All animal procedures were conducted in accordance with the Canadian Council on Animal Care guidelines and approved by the Queen's University Animal Care Committee.

### FVIII Concentrates

rFVIIIFc, yellow fluorescent protein—tagged (YFP) rFVIIIFc and BDD FVIII were expressed and purified as previously described (22). For the production of YFP rFVIIIFc, the YFP sequence was inserted in place of the B domain within the rFVIIIFc construct. Similarly, for the production of BDD FVIII the Fc sequence was removed from the rFVIIIFc construct. All concentrates had similar specific activity of 8,000–10,000 IU/ mg and were a kind gift from Bioverativ, a Sanofi company.

### Cells

FVIII-exposed whole splenocytes were generated by harvesting spleens from FVIII-exposed hemophilia A mice 1 week after their last FVIII injection. Naïve whole splenocytes were generated by harvesting spleens from sex and age matched hemophilia A mice that had not been exposed to FVIII.

In order to generate naïve and FVIII-exposed B cells, whole splenocytes from naïve and FVIII-exposed mice were first subjected to red blood cell lysis followed by negative selection using the EasySep mouse B cell isolation kit (Stem Cell Technologies). Cells from multiple mice (~3–5) were pooled to generate FVIII-exposed and naïve B cell fractions.

Some experiments were repeated using 413 cells, a murine B cell hybridoma that expresses anti-FVIII A2 IgG1 (23). These cells were characterized for receptors of interest via flow cytometry using Alexa Fluor 488 anti-IgG (Invitrogen), APC anti-FcγRIIB and FITC anti-CD79a (eBiosciences).

### rFVIIIFc Binding Assay

Whole splenocytes from naïve or FVIII-exposed mice as well as 413 cells were incubated with varying doses of BDD FVIII (0, 0.1, 0.2, and 0.4 μg/test) or APC-conjugated anti-FcγRIIB (APC anti-FcγRIIB: 0, 0.1, 0.2, and 0.4 μg/test) for 30 min at 4°C in order to block potential binding sites of rFVIIIFc on these cells. Anti-FcγRIIB antibody clone AT130-2 was used because it has previously been shown to have agonistic effects against its target (24) and prevent binding of FVIII immune complexes to FcγRIIB (19). YFP rFVIIIFc was then added at 0.3 μg/test for 30 min at 4°C. The amount of YFP rFVIIIFc binding was then measured via flow cytometry (SH800S, Sony). To identify the B cell subset of the whole splenocyte suspension a PE-Cy7-conjugated CD19 (PE-Cy7 CD19) antibody was used (BD Pharmingen).

### Western Blots

Naïve and FVIII-exposed B cells as well as 413 cells were incubated with BDD FVIII (11.4 μg/ml), rFVIIIFc (14.7 μg/ml), goat anti-mouse IgG F(ab)_2_ (αIgG F(ab)_2_, 20 μg/ml, Southern Biotech) or whole goat anti-mouse IgG (αIgG, 20 μg/ml, Southern Biotech) for 30 min at 37°C. Cell lysates were then extracted and separated on an SDS PAGE gel, followed by transfer to nitrocellulose membrane (Bio Rad). Membranes were then blotted for phosphorylated SH2-containing inositol phosphatase (pSHIP, Cell Signaling Technology), SHIP (Santa Cruz Biotechnology), phosphorylated ERK (pERK, Cell Signaling Technology), ERK (Cell Signaling Technology) and actin (Abcam). Detection was carried out using horseradish peroxidase—conjugated (HRP) goat anti-rabbit (Dako) and goat anti-mouse (Southern Biotech) Ig followed by development with an enhanced chemiluminescence substrate (PerkinElmer). Densitometry analysis was performed using ImageJ (NIH) and ratios of phosphorylated to total protein were averaged for three different blots. No statistical analysis was carried out for these data due to the qualitative nature of the assay.

### Calcium Flux Assay

Whole splenocytes from naïve hemophilia A mice were stained with 2.6 μM Fluo-3 (Invitrogen) and 5.5 μM Fura Red (Invitrogen) for 45 min at 37°C. To identify the B cell subset of the whole splenocytes suspension a PE-Cy7 CD19 antibody was used (BD Pharmingen). B cell calcium flux was then assessed using flow cytometry (SH800S, Sony). Following 5 min of baseline fluorescence reading, αIgG (10 μg/ml, Southern Biotech), αIgG F(ab)_2_ (10 μg/ml, Southern Biotech), αIgG F(ab)_2_ + BDD FVIII (11.4 μg/ml) or αIgG F(ab)_2_ + rFVIIIFc (14.7 μg/ml) were added and data were acquired for a further 7 min. All samples were then treated with ionomycin (1.4 μM) to elicit a maximal response and then finally quenched with EGTA (5 mM). Data was then analyzed using FlowJoX (Tree Star) and the median ratio of Fluo-3 to Fura Red fluorescence was reported as a measure of intracellular calcium flux.

### Statistics

All binding competition assays were compared using a 1-way ANOVA followed by Tukey's multiple comparison test. For the competition with anti-FcγRIIB, the percentage of rFVIIIFc^+^, rFVIIIFc^+^FcγRIIB^+^, or FcγRIIB^+^ cells at 0.2 and 0.4 μg of block were compared against the same parameter at 0.1 μg of block. For the competition with FVIII, the percentage of rFVIIIFc^+^ cells at all block doses was compared against the same parameter at baseline (0 μg block). Statistical analyses were performed using GraphPad Prism 5.0a (GraphPad Software).

## Results

### rFVIIIFc Binds the FcγRIIB of Naïve and FVIII-Exposed B Cells and Splenocytes

**Figure 1:**
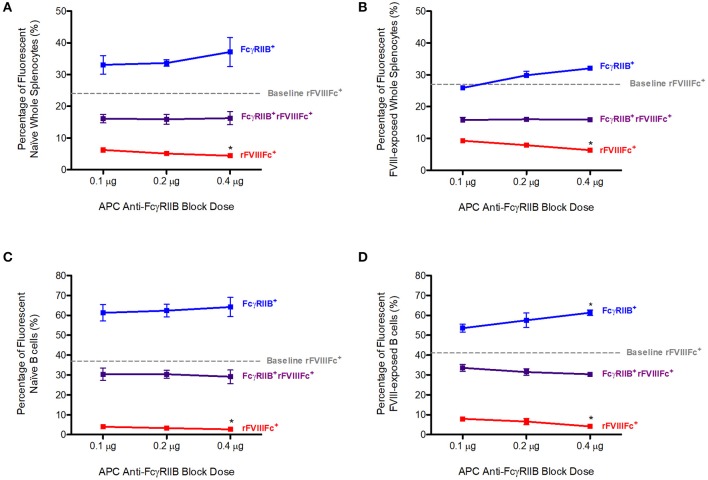
Competition with anti-FcγRIIB decreases rFVIIIFc binding to naïve and FVIII-exposed splenocytes and B cells. The percentage of rFVIIIFc^+^, FcγRIIB^+^rFVIIIFc^+^ and FcγRIIB^+^ **(A)** naïve whole splenocytes, **(B)** FVIII-exposed whole splenocytes, **(C)** naïve B cells, **(D)** FVIII-exposed B cells when blocking with APC anti-FcγRIIB (0.1, 0.2, or 0.4 μg) prior to YFP rFVIIIFc (0.3 μg) incubation. Baseline rFVIIIFc corresponds to the percentage of rFVIIIFc^+^ cells in the absence of APC anti-FcγRIIB. Statistical analysis compares the percentage of rFVIIIFc^+^, rFVIIIFc^+^FcγRIIB^+^, or FcγRIIB^+^ cells at 0.2 and 0.4 μg of block against the same parameter at 0.1 μg of block. *n* = 3/condition. Error bars represent SD. *p < 0.05.

Naïve or FVIII-exposed whole splenocytes were first incubated with 0.1, 0.2, or 0.4 μg of APC anti-FcγRIIB antibody. Following this, 0.3 μg of YFP rFVIIIFc was added to each sample. The percentage of rFVIIIFc^+^ cells in the absence of APC anti-FcγRIIB (0 μg) was determined to be the baseline level of rFVIIIFc binding to these cells. This corresponded with 24% of naïve and 27% of FVIII-exposed whole splenocytes (Figures 1A,B). Blocking of these cells with anti-FcγRIIB prior to YFP rFVIIIFc exposure was able to significantly decrease YFP rFVIIIFc binding to both naïve (*p* = 0.0478, Figure 1A) and FVIII-exposed (*p* = 0.0036, Figure 1B) whole splenocytes in a dose-dependent manner. In this experiment we also observed a number of cells positive for both FcγRIIB and rFVIIIFc (rFVIIIFc^+^FcγRIIB^+^). The percentage of rFVIIIFc^+^FcγRIIB^+^ double positive B cells remained constant across the varying doses of anti-FcγRIIB block and indicates that rFVIIIFc does not interact with these cells solely through FcγRIIB. Representative raw flow cytometry data can be found in the Supplementary Materials.

By adding a PE-Cy7 anti-CD19 antibody to the whole splenocytes suspensions, we were also able to investigate the interaction of rFVIIIFc with B cells. The baseline rFVIIIFc binding to naïve and FVIII-exposed B cells corresponded to 37 and 41%, respectively (Figures 1C,D). Once again, in the presence of increasing doses of APC anti-FcγRIIB, YFP rFVIIIFc binding to naïve (*p* = 0.0478, Figure 1C) and FVIII-exposed (*p* = 0.0084, Figure 1D) B cells decreased in a dose-dependent manner. This effect was more pronounced in B cells than whole splenocytes. rFVIIIFc^+^FcγRIIB^+^ double positive B cells showed a similar pattern to the one observed with whole splenocytes. Representative raw flow cytometry data can be found in the Supplementary Materials.

Together these data indicate that rFVIIIFc is able to bind naïve and FVIII-exposed splenocytes and B cells via FcγRIIB. However, since we also observed a significant percentage of rFVIIIFc^+^FcγRIIB^+^ cells it is likely that rFVIIIFc has additional modes of interaction with these cells.

### rFVIIIFc Binds the BCR of Naïve and FVIII-Exposed B Cells and Splenocytes

**Figure 2:**
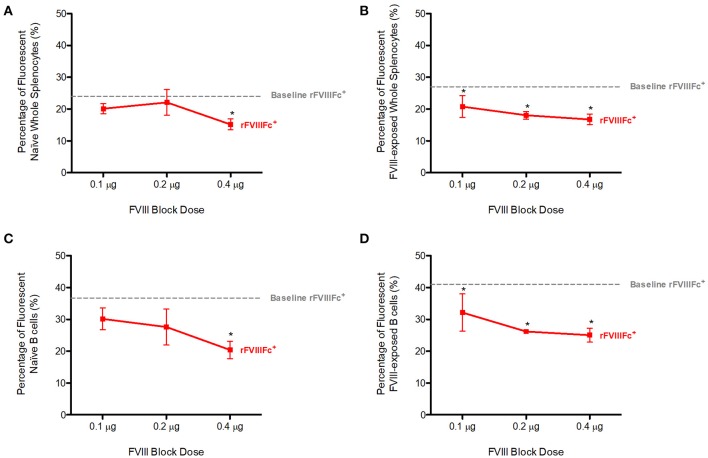
Competition with FVIII decreases rFVIIIFc binding to naïve and FVIII-exposed splenocytes and B cells. The percentage of rFVIIIFc^+^ **(A)** naïve whole splenocytes, **(B)** FVIII-exposed whole splenocytes, **(C)** naïve B cells, **(D)** FVIII-exposed B cells when blocking with FVIII (0.1, 0.2, or 0.4 μg) prior to YFP rFVIIIFc (0.3 μg) incubation. Baseline rFVIIIFc corresponds to the percentage of rFVIIIFc^+^ cells in the absence of FVIII. Statistical analysis compares the percentage of rFVIIIFc^+^ cells at all block doses against the same parameter at 0 μg block. *n* = 3/condition. Error bars represent SD. *p < 0.05.

We repeated the previous experiment using FVIII as a block instead of APC anti-FcγRIIB. Pre-blocking with FVIII was able to significantly decrease YFP rFVIIIFc binding to both naïve (*p* = 0.0019, Figure 2A) and FVIII-exposed (*p* = 0.0150, Figure 2B) whole splenocytes in a dose-dependent manner.

When looking at the B cell compartment, once again in the presence of increasing doses of FVIII, YFP rFVIIIFc binding to naïve (*p* = 0.0200, Figure 2C) and FVIII-exposed (*p* = 0.0013, Figure 2D) B cells decreased in a dose-dependent manner. This effect was more pronounced in FVIII-exposed whole-splenocytes and B cells than their naïve counterparts.

We therefore concluded that FVIII blocks rFVIIIFc binding to naïve and FVIII-exposed splenocytes and B cells. Although multiple mechanisms might explain interactions between FVIII and these cells, our observations can in part be attributed to FVIII BCR binding.

### rFVIIIFc Affects Signaling in Both Naïve and FVIII-Exposed Splenocytes

**Figure 3:**
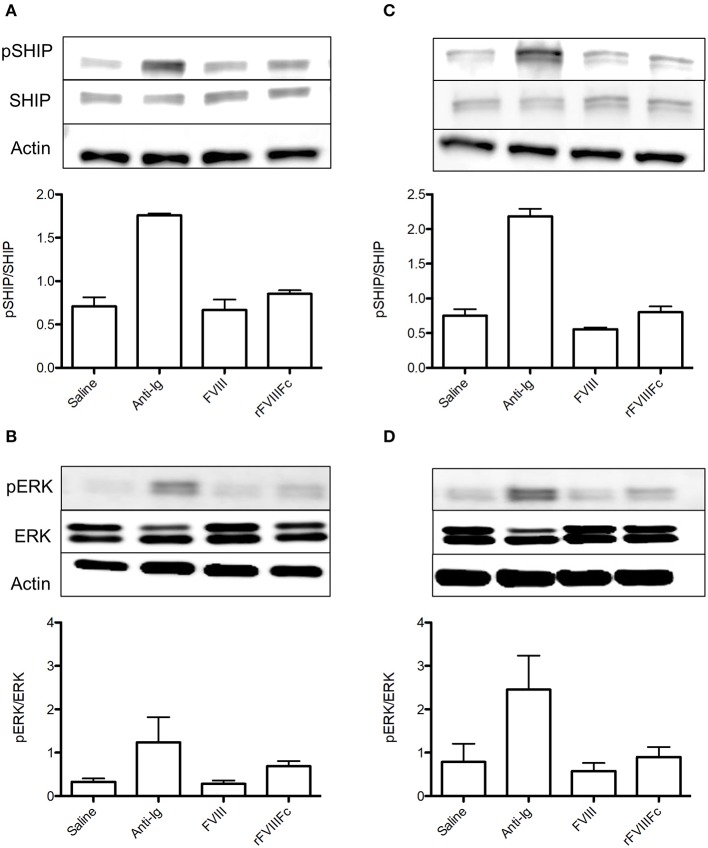
rFVIIIFc affects signaling in both naïve and FVIII-exposed whole splenocytes. pSHIP and pERK levels in saline, anti-Ig (20 μg/ml), FVIII (11.4 μg/ml), and rFVIIIFc (14.7 μg/ml) stimulated **(A,B)** naïve and **(C,D)** FVIII-exposed splenocytes. Ratios of phosphorylated to total protein were obtained through densitometry analysis of three different blots. *n* = 3/condition. Error bars represent SD.

We next sought to investigate the ability of rFVIIIFc binding to influence immune cell signaling. Naïve and FVIII-exposed whole splenocytes were incubated with saline, anti-Ig, FVIII or rFVIIIFc for 30 min. We then assessed the effect of these agents on SHIP and ERK phosphorylation, two key mediators of the FcγRIIB and BCR signaling pathways. The inhibitory signals induced by cross-linking these two receptors have been shown to rely on SHIP phosphorylation (14). In both naïve and FVIII-exposed whole splenocytes rFVIIIFc resulted in increased SHIP phosphorylation when compared to FVIII (Figures 3A,C). This was also accompanied by increased ERK phosphorylation (Figures 3B,D), which is typically associated with the propagation of activating signals through both the BCR and other cell surface receptors (25). These findings therefore suggest that rFVIIIFc affects cell signaling of both naïve and FVIII-exposed splenocytes. However, based solely on these experiments it cannot be determined if the overall net effect results in activation or inhibition of these cells.

### rFVIIIFc Induces Inhibitory Signaling in FVIII-Exposed but Not Naïve B Cells

**Figure 4:**
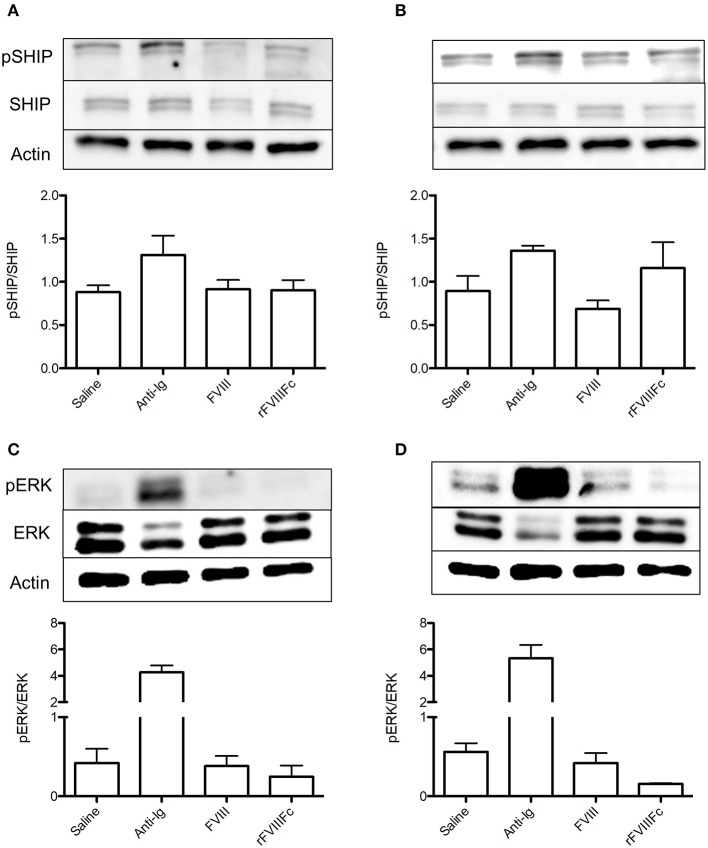
rFVIIIFc induces inhibitory signaling in FVIII-exposed but not naïve B cells. pSHIP and pERK levels in saline, anti-Ig (20 μg/ml), FVIII (11.4 μg/ml), and rFVIIIFc (14.7 μg/ml) stimulated **(A,B)** naïve and **(C,D)** FVIII-exposed B cells. Ratios of phosphorylated to total protein were obtained through densitometry analysis of three different blots. *n* = 3/condition. Error bars represent SD.

In order to isolate the effect of rFVIIIFc on the B cell compartment, we repeated the aforementioned experiment using naïve and FVIII-exposed B cells. In naïve B cells, rFVIIIFc and FVIII had comparable effects on the levels of SHIP phosphorylation (Figure 4A). This was accompanied by a minimal decrease in ERK phosphorylation in the presence of rFVIIIFc (Figure 4B). Together these results suggest that rFVIIIFc does not significantly impact naïve B cell signaling. However, when these studies were repeated using FVIII-exposed B cells, rFVIIIFc resulted in increased SHIP phosphorylation and decreased ERK phosphorylation (Figures 4C,D) when compared to FVIII. rFVIIIFc can therefore selectively induce inhibitory signaling in FVIII-exposed B cells.

### rFVIIIFc Inhibits Anti-Ig F(ab)_2_ Induced Calcium Flux in B Cells

**Figure 5:**
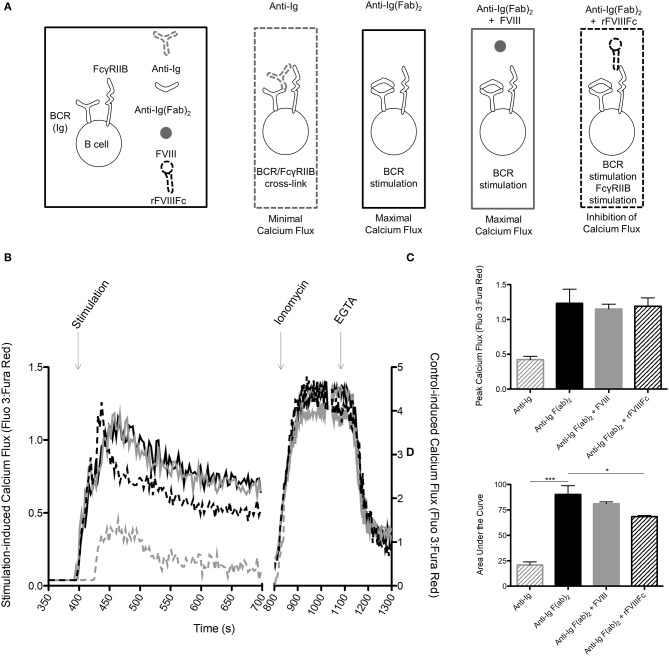
rFVIIIFc inhibits anti-Ig F(ab)_2_ induced calcium flux in B cells. **(A)** Calcium flux assay stimulation conditions and their hypothesized outcomes. **(B)** Representative graph of calcium flux assay results. **(C)** Peak calcium flux and **(D)** area under the curve for B cells stimulated with anti-Ig (10 μg/ml), anti-Ig F(ab)_2_ (10 μg/ml), anti-Ig F(ab)_2_ + FVIII (10 + 11.4 μg/ml) and anti-Ig F(ab)_2_ + rFVIIIFc (10 + 14.7 μg/ml). *n* = 3/condition. Errors bars represent SD. *p < 0.05, ***p < 0.001. 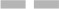, anti-Ig; 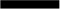, anti-Ig F(ab)_2_; 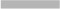, anti-Ig F(ab)_2_ + FVIII; 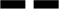, anti-Ig F(ab)_2_ + rFVIIIFc.

We next sought to determine if rFVIIIFc is able to inhibit B cell calcium flux: a hallmark of BCR stimulation and B cell activation. Calcium flux assays are only able to detect pan-B cell stimulation and are not sensitive enough to detect changes induced by a specific antigen. In accordance with this fact, we could not detect the effect of FVIII or rFVIIIFc on FVIII-exposed B cell calcium flux. Instead, we opted to investigate the ability of these proteins to inhibit non-specific B cell stimulation induced by anti-Ig F(ab)_2_. Using B cells from hemophilia A mice, we first measured the calcium flux induced by anti-Ig F(ab)_2_ and anti-Ig to determine the maximal and minimal responses. We then assessed the calcium flux induced by anti-Ig F(ab)_2_ in these cells in the presence of FVIII (anti-Ig F(ab)_2_ + FVIII) or rFVIIIFc (anti-Ig F(ab)_2_ + rFVIIIFc) (Figure 5A). When stimulated with anti-Ig F(ab)_2_ B cells reached an average peak flux of 1.23 with an average area under the curve (AUC) of 90.2 (Figures 5B–D). As expected, in the presence of intact anti-Ig these cells had a significantly blunted calcium response (peak = 0.42, AUC = 20.8, Figures 5B–D), indicative of cross-linking the BCR with FcγRIIB. When incubated with anti-Ig F(ab)_2_ + FVIII, B cells showed a similar calcium flux profile to the one observed in the presence of anti-Ig F(ab)_2_ alone (peak = 1.15, AUC = 81.1, Figures 5B–D). Although anti-Ig F(ab)_2_ + rFVIIIFc cells reached a similar peak calcium flux of 1.15, they had an overall attenuated response as indicated by the smaller AUC of 68.3 (Figures 5B–D). This demonstrates that in the presence of rFVIIIFc the influx of calcium typically caused by anti-Ig F(ab)_2_ is decreased, suggesting an inhibitory effect of rFVIIIFc on B cell activation.

### 413 Cells Are an Appropriate Model for Assessing rFVIIIFc Binding and FcγRIIB Signaling

A significant challenge of the experiments described thus far is the low frequency of FVIII-specific B cells within the B cell compartment isolated from even the FVIII-exposed mice. This not only required several animals to generate sufficient reagents, but also resulted in small differences between the FVIII and rFVIIIFc groups, requiring sensitive assays. We were therefore interested in exploring a clonal B cell with FVIII-specificity as an alternative model.

**Figure 6:**
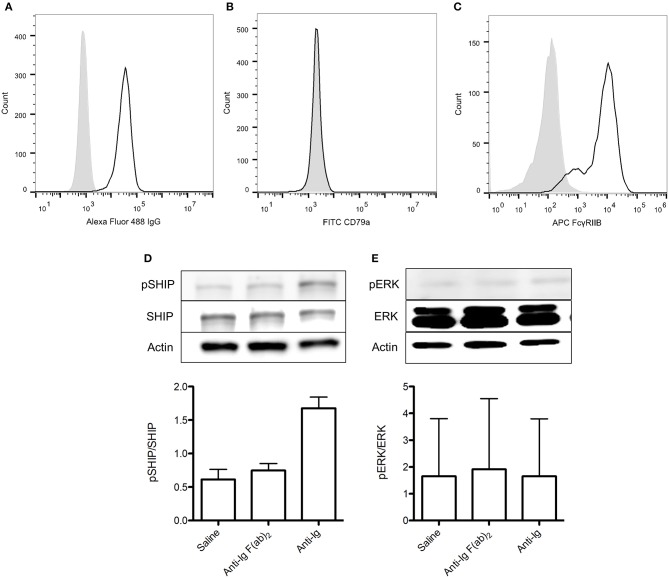
413 cells are an appropriate model for assessing rFVIIIFc binding and FcγRIIB signaling. **(A)** IgG, **(B)** CD79a, and **(C)** FcγRIIB expression of 413 cells. **(D)** pSHIP and **(E)** pERK levels in 413 cells stimulated with saline, anti-Ig F(ab)_2_ (20 μg/ml) and anti-Ig (20 μg/ml). Ratios of phosphorylated SHIP to total SHIP were obtained through densitometry analysis of three different blots. *n* = 3/condition. Error bars represent SD. 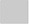, control isotype antibody; 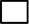, antibody of interest.

As previously described, the 413 cell line is a murine B cell hybridoma that expresses anti-FVIII A2 domain IgG1 (23). To assess the appropriateness of using this cell type in our experiments we first characterized the expression of surface IgG and FcγRIIB on these cells via flow cytometry. We also assessed their intracellular expression of CD79a, which is required for transduction of positive IgG signaling. Although 413 cells expressed both IgG and FcγRIIB, they lacked CD79a expression (Figures 6A–C). As such, they would only be appropriate for investigating the ability of rFVIIIFc to signal via FcγRIIB rather than both the BCR and FcγRIIB. To confirm this conclusion, we stimulated these cells with saline, anti-Ig F(ab)_2_ and anti-Ig. As expected, anti-Ig was able to induce SHIP phosphorylation via engagement of FcγRIIB (Figure 6D). Furthermore, anti-Ig F(ab)_2_ did not induce ERK phosphorylation which would have indicated the transduction of activating signals through the BCR (Figure 6E). We therefore concluded that 413 cells could only be used to assess the ability of rFVIIIFc to engage and signal through FcγRIIB.

### rFVIIIFc Binds 413 Cells via FcγRIIB as Well as the BCR and Results in Increased SHIP Phosphorylation

**Figure 7:**
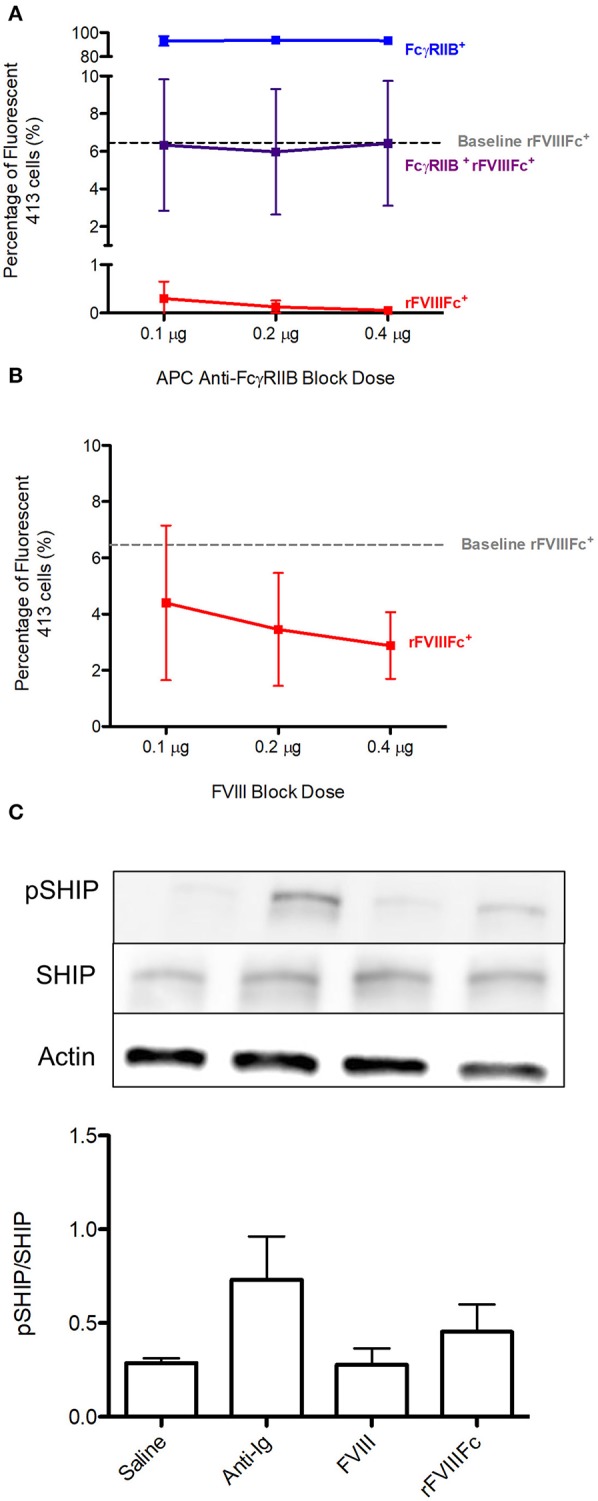
rFVIIIFc binds 413 cells via FcγRIIB as well as the BCR and results in increased SHIP phosphorylation. The percentage of rFVIIIFc^+^ 413 cells when blocking with **(A)** APC anti-FcγRIIB (0.1, 0.2, or 0.4 μg) or **(B)** FVIII (0.1, 0.2, or 0.4 μg) prior to YFP rFVIIIFc (0.3 μg) incubation. Baseline rFVIIIFc corresponds to the percentage of rFVIIIFc^+^ cells in the absence of APC anti-FcγRIIB or FVIII. **(C)** pSHIP levels in saline, anti-Ig (20 μg/ml), FVIII (11.4 μg/ml) and rFVIIIFc (14.7 μg/ml) stimulated 413 cells. Ratios of phosphorylated SHIP to total SHIP were obtained through densitometry analysis of three different blots. *n* = 3/condition. Error bars represent SD.

Using 413 cells, we repeated the binding experiments investigating the ability of rFVIIIFc to interact with FcγRIIB and the BCR. The baseline rFVIIIFc binding to 413 cells was 6% (Figures 7A,B). Once again, both anti-FcγRIIB and FVIII inhibited binding of rFVIIIFc to these cells (Figures 7A,B). When looking at the downstream effects of rFVIIIFc binding to 413 cells, an increase in SHIP phosphorylation was observed, providing further proof of rFVIIIFc's ability to induce inhibitory signaling via FcγRIIB (Figure 7C).

## Discussion

The aim of these experiments was to investigate the ability of rFVIIIFc to inhibit activation of FVIII-specific B cells by cross-linking their BCR with the inhibitory FcγRIIB receptor. We demonstrate that rFVIIIFc can bind naïve and FVIII-exposed B cells. Blockade with an anti-FcγRIIB antibody or FVIII resulted in decreased rFVIIIFc binding to these cells, suggesting that FcγRIIB and FVIII-specific BCR both play a role in these interactions. The incomplete blockade of rFVIIIFc binding by either of these agents and the presence of rFVIIIFc^+^FcγRIIB^+^ double positive cells indicates that rFVIIIFc binding to B cells is not solely mediated by these receptors. Other B cell surface receptors, such as Siglec-5, have been shown to bind FVIII (26). In addition, non-specific membrane binding through the phospholipid-binding motif of the FVIII C2 domain may also be playing a role in this finding (27). Finally, there may be yet unidentified binding partners for rFVIIIFc facilitating interactions of this protein with B cells.

rFVIIIFc was also able to induce inhibitory signaling in FVIII-exposed B cells as indicated by increased SHIP and decreased ERK phosphorylation. These changes were not observed in naïve B cells suggesting that the inhibitory effects of rFVIIIFc are limited to FVIII-specific B cells. When compared to the positive control (anti-Ig) the effect of rFVIIIFc on B cell signaling appears to be quite modest. While anti-Ig is able to engage all B cells regardless of their specificity, the frequency of cells able to respond to FVIII or rFVIIIFc is small and thus a reduced inhibitory effect is expected (28).

rFVIIIFc binding also occurred in the setting of naïve and FVIII-exposed whole splenocytes. Although this resulted in altered signaling when compared to FVIII, the overall effect on these cells was unclear. This is likely due to the heterogeneous cell population and the ubiquitous expression of Fc receptors. Thus far, rFVIIIFc has been shown to affect regulatory T cells (16) and macrophages (29), both of which can be found in the spleen. However, it is likely that it has a number of other cellular interactions that are yet to be characterized and which could account for our findings. In addition to its role in BCR and FcγRIIB signaling, SHIP is involved in skewing T cell responses and driving macrophage maturation (30). Similarly, ERK is involved in the signal transduction of many mitogens including activators of the BCR and TCR (31). Initiation of any of these pathways would have therefore been detected by our assays complicating the interpretation of the results.

Cross-linking of the BCR with FcγRIIB has also been associated with inhibition of B cell calcium flux. In our studies, rFVIIIFc was able to attenuate calcium flux in B cells stimulated with anti-Ig F(ab)_2_ more effectively than FVIII. Although both of these molecules resulted in a similar peak calcium flux, rFVIIIFc was associated with a decreased AUC, which indicates a dampened calcium response. Due to its limited sensitivity, a Fluo 3: Fura Red assay can only detect calcium fluxes induced by pan-B cell stimulation rather than single antigens and so we were unable to detect the isolated effect of FVIII or rFVIIIFc (32). Instead, we opted to investigate the ability of these molecules to inhibit calcium flux induced by anti-Ig F(ab)_2_ stimulation. The experimental set-up also required that anti-Ig F(ab)_2_ and FVIII or rFVIIIFc were added to the sample sequentially. This may have affected the peak calcium fluxes that were observed as cells were not exposed to the activating and inhibitory reagents simultaneously. It may also explain why rFVIIIFc did not attenuate B cell signaling to the same degree as anti-Ig. Despite these challenges, the ability of rFVIIIFc to dampen the calcium flux induced by a potent pan-BCR stimulant is apparent and encouraging.

A recurrent obstacle for both this and other studies evaluating the responses of FVIII-specific B cells is the small size of this cellular subset. As an alternative to using primary cells from mice exposed to FVIII we explored the use of 413 cells as a clonal model of FVIII-specific B cells. Although this mouse B cell hybridoma expressed BCR and FcγRIIB in abundance, it lacked CD79a expression, resulting in an inability to generate activating BCR-induced signaling. We therefore deemed this model appropriate to use when investigating rFVIIIFc binding and FcγRIIB signaling in isolation, but not dual signaling through both the BCR and FcγRIIB. In the future, methods to generate stable FVIII-specific B cell lines or expand the number of these cells from a primary source would be of great benefit to assess therapeutic effects of FVIII B cell contributions.

Although rFVIIIFc was able to bind 413 cells, it did so to a surprisingly low degree considering that virtually all cells expressed BCR and FcγRIIB. It is however important to note that the BCR of these cells is specific for the A2 domain and so the avidity of these cells for FVIII is lower than in a polyclonal B cell population. In addition, because this is a hybridoma cell line, the surface BCR expression of 413 cells is likely transient rather than stable. These factors may therefore interfere with rFVIIIFc binding to the BCR. Physiologically, FcγRIIB typically binds the Fc of immune complexed IgG with low affinity. In the setting of monomeric Fc, its binding affinity is even lower. It may therefore be difficult to capture interactions between these two molecules.

Throughout these experiments we used equimolar concentrations of rFVIIIFc (~15 μg/ml), BDD FVIII (~11 μg/ml), and anti-Ig (~10 μg/ml). These doses correspond to FVIII concentrations of ~100 IU/ml and were consistent with those previously shown to result in B cell inhibition *in vitro* (1). Hemophilia A patients with inhibitors undergoing even the most aggressive ITI protocols receive 200 IU/kg/day of FVIII which, for an average sized adult male, is equivalent to about 2.8 IU/ml. Doses required for B cell inhibition may therefore not be achievable in patients. That being said, the kinetics of the interactions between rFVIIIFc and B cells are likely drastically different *in vivo*. It is therefore difficult to determine if the same rFVIIIFc dosing would be required to reproduce the findings of our studies in the context of clinical practice.

All of our experiments were carried out in the absence of pre-formed anti-FVIII antibodies, which would be expected in the setting of a hemophilia A patient with inhibitors. Since IgG4 is the isotype most commonly associated with inhibitory activity, it is reasonable to hypothesize that during ITI, FVIII/IgG4 immune complexes are formed. This isotype is similar to IgG1 in its affinity for FcγRIIB (33). The potential role of BCR and FcγRIIB co-engagement by FVIII/IgG4 immune complexes in the mechanism of ITI should therefore be investigated. FcγRIIB is known to have a higher affinity for immune complexes than singly IgG-bound antigen. Due to its Fc component, rFVIIIFc may form immune complexes of large-enough size more readily than conventional FVIII. Our findings may also provide a further mechanistic basis for the decreased immunogenicity of rFVIIIFc documented in pre-clinical models (16).

Based on the molecular findings presented here and the limited clinical evidence available thus far, rFVIIIFc may have improved ITI performance when compared to conventional FVIII. This could represent a significant improvement for hemophilia A patients with inhibitors by decreasing the length of therapy and the number of infusions required to achieve immunologic tolerance. It could also decrease health care costs by not only shortening ITI duration but also avoiding complications associated with the delay or failure to achieve tolerance (e.g., bleeding, arthropathy). Current approaches to improving ITI performance require the use of immunosuppressive reagents that have generalized off-target effects. In contrast, rFVIIIFc could improve ITI efficacy with the added benefit of maintaining antigen specificity.

## Conclusions

The work we present here demonstrates that rFVIIIFc binds naïve and FVIII-exposed B cells. These interactions can be inhibited by blockade with anti-FcγRIIB and FVIII indicating that rFVIIIFc can engage FcγRIIB as well as the BCR of these cells. FVIII-exposed B cells incubated with rFVIIIFc exhibited increased SHIP phosphorylation and decreased ERK phosphorylation when compared to those incubated with FVIII. These effects were not observed in naïve B cells. Furthermore, rFVIIIFc was able to decrease the magnitude of calcium flux induced by pan-B cell stimulation using anti-Ig F(ab)_2_. Together, these data show that rFVIIIFc can inhibit B cell signaling in an antigen-specific matter. These findings provide a potential molecular mechanism for the improved performance of rFVIIIFc in the context of ITI, and support the use of this concentrate as an alternative to conventional FVIII to achieve a quicker time to tolerance induction.

## Data Availability Statement

All datasets generated for this study are included in the article/Supplementary Material.

## Ethics Statement

All animal studies were reviewed and approved by the Queen's University Animal Care Committee.

## Author Contributions

MG designed, performed experiments, analyzed data, and wrote the manuscript. PM, JD, CH, and DL designed experiments, edited, and approved the manuscript. TL performed experiments. DS provided reagents, edited, and approved the manuscript.

### Conflict of Interest

DL receives research funding from Bayer, Biomarin, Bioverativ/Sanofi, CSL-Behring and Octapharma. TL and JD are employees of Bioverativ, a Sanofi company. The remaining authors declare that the research was conducted in the absence of any commercial or financial relationships that could be construed as a potential conflict of interest. The reviewer SD and handling editor declared their shared affiliation at the time of review.
