# Supplementary material for: Recombinant Factor VIII Fc Inhibits B Cell Activation via Engagement of the FcγRIIB Receptor
Source: Front Immunol. 2020 Feb 7;11:138. doi: 10.3389/fimmu.2020.00138 (PMC7025534; doi:10.3389/fimmu.2020.00138)
Supplement: Supplementary file 1 [file Data_Sheet_1.PDF]

Supplementary Material

1 Supplementary Figures

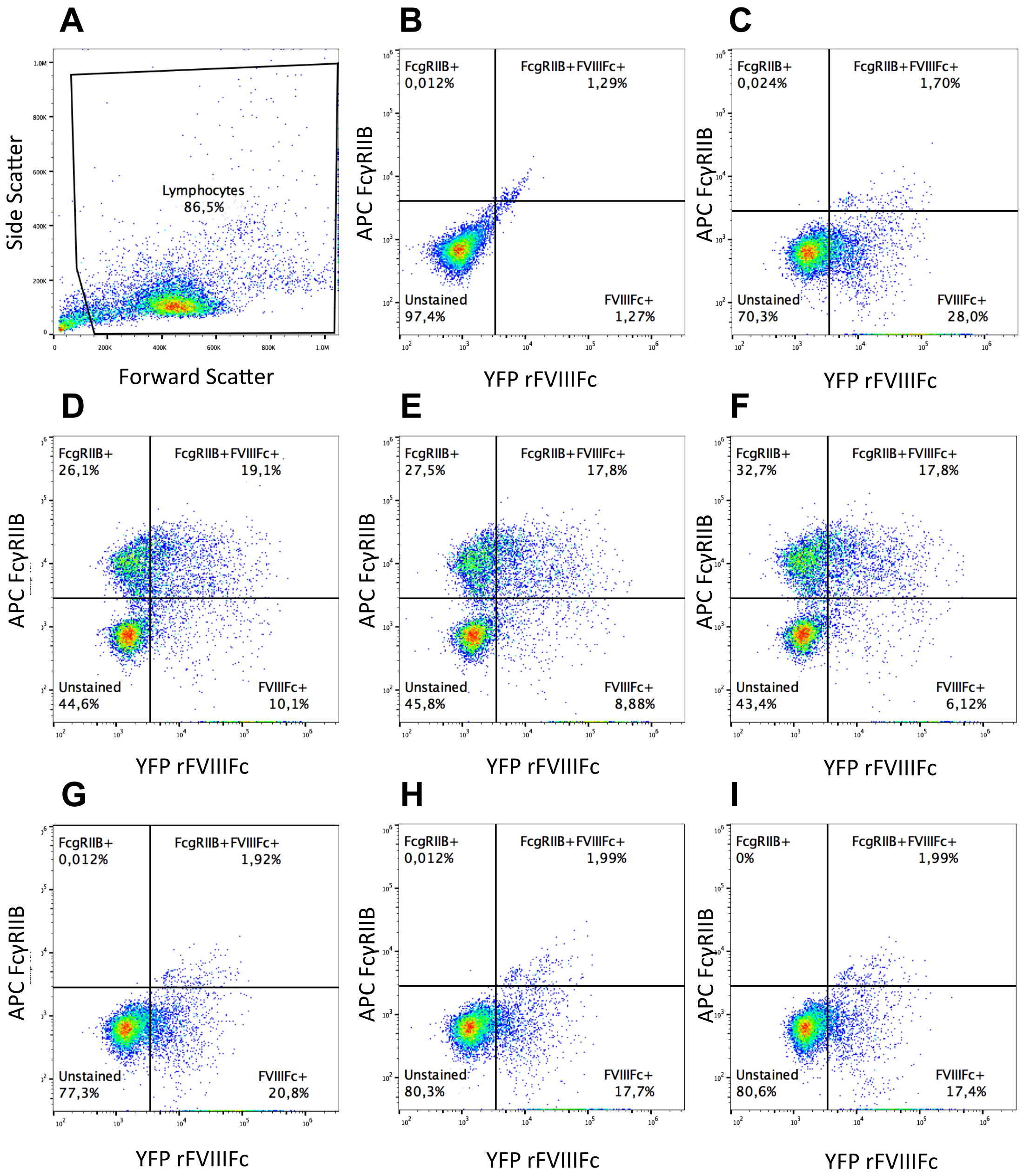

Supplementary Figure 1. Representative flow cytometry results of the rFVIIIFc binding assay with FVIII-exposed splenocytes. (A-B) Unstained control. FVIII-exposed lymphocytes stained with **(C)** YFP rFVIIIFc, **(D)** 0.4 µg APC anti-FcγRIIB + YFP rFVIIIFc, **(E)** 0.2 µg APC anti-FcγRIIB + YFP rFVIIIFc, **(F)** 0.1 µg APC anti-FcγRIIB + YFP rFVIIIFc, **(G)** 0.4 µg FVIII + YFP rFVIIIFc, **(H)** 0.2 µg FVIII + YFP rFVIIIFc, **(I)** 0.1 µg FVIII + YFP rFVIIIFc.
YFP = yellow fluorescent protein.

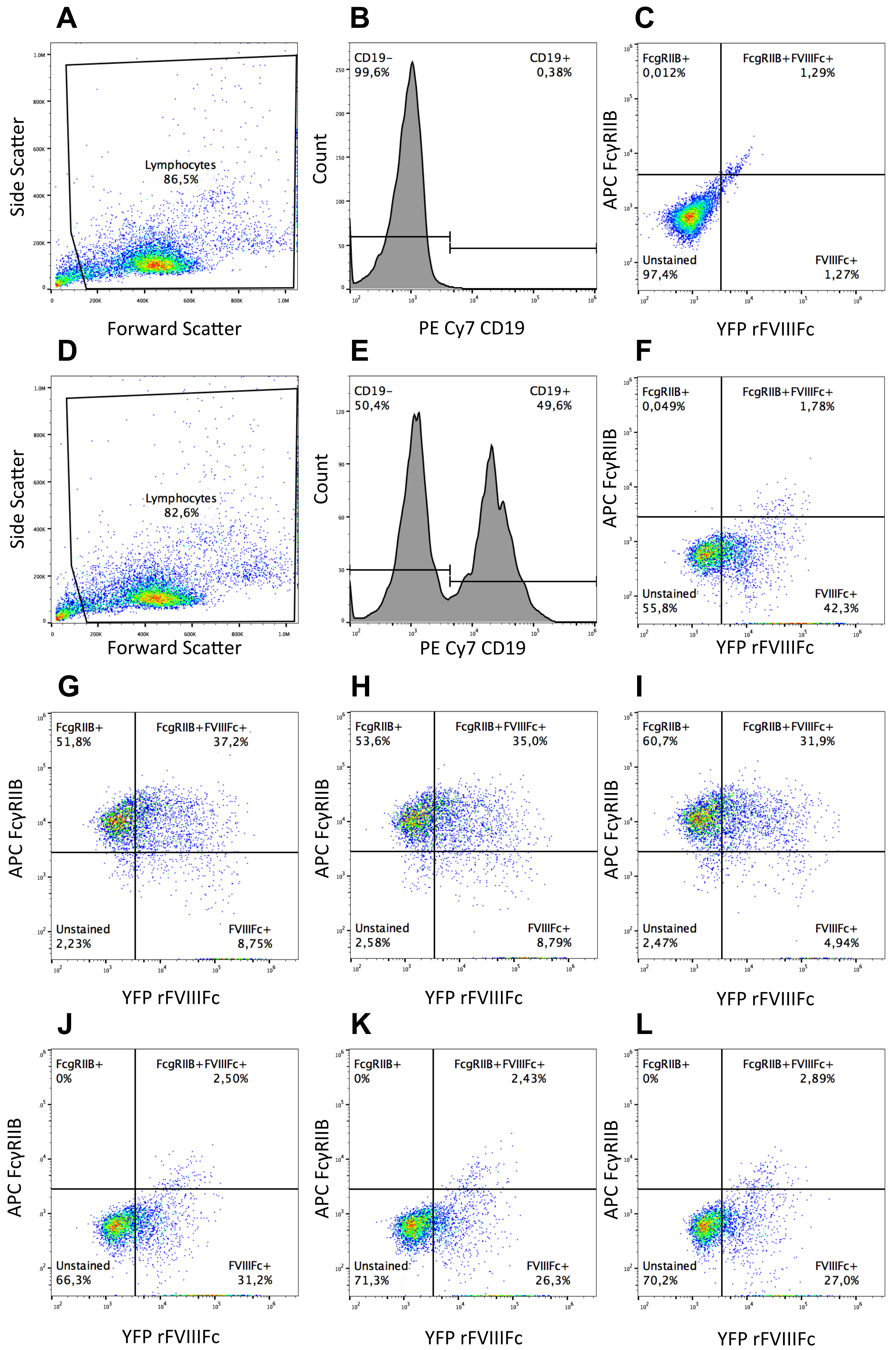
A
Side Scatter
Lymphocytes
86,5%
Forward Scatter
B
Count
CD19−
99,6%
CD19+
0,38%
PE Cy7 CD19
C
APC FcγRIIB
FcgRIIB+
0,012%
FcgRIIB+FVIIIFc+
1,29%
Unstained
97,4%
FVIIIFc+
1,27%
YFP rFVIIIFc
D
Side Scatter
Lymphocytes
82,6%
Forward Scatter
E
Count
CD19−
50,4%
CD19+
49,6%
PE Cy7 CD19
F
APC FcγRIIB
FcgRIIB+
0,049%
FcgRIIB+FVIIIFc+
1,78%
Unstained
55,8%
FVIIIFc+
42,3%
YFP rFVIIIFc
G
APC FcγRIIB
FcgRIIB+
51,8%
FcgRIIB+FVIIIFc+
37,2%
Unstained
2,23%
FVIIIFc+
8,75%
YFP rFVIIIFc
H
APC FcγRIIB
FcgRIIB+
53,6%
FcgRIIB+FVIIIFc+
35,0%
Unstained
2,58%
FVIIIFc+
8,79%
YFP rFVIIIFc
I
APC FcγRIIB
FcgRIIB+
60,7%
FcgRIIB+FVIIIFc+
31,9%
Unstained
2,47%
FVIIIFc+
4,94%
YFP rFVIIIFc
J
APC FcγRIIB
FcgRIIB+
0%
FcgRIIB+FVIIIFc+
2,50%
Unstained
66,3%
FVIIIFc+
31,2%
YFP rFVIIIFc
K
APC FcγRIIB
FcgRIIB+
0%
FcgRIIB+FVIIIFc+
2,43%
Unstained
71,3%
FVIIIFc+
26,3%
YFP rFVIIIFc
L
APC FcγRIIB
FcgRIIB+
0%
FcgRIIB+FVIIIFc+
2,89%
Unstained
70,2%
FVIIIFc+
27,0%
YFP rFVIIIFc

Supplementary Figure 2. Representative flow cytometry results of the rFVIIIFc binding assay with FVIII-exposed B cells. **(A-B)** Unstained lymphocytes. **(C)** Unstained CD19+ FVIII-exposed lymphocytes. **(D-E)** FVIII-exposed lymphocytes stained with PE Cy7 CD19. CD19+ FVIII-exposed lymphocytes stained with **(F)** YFP rFVIIIFc, **(G)** 0.4 µg APC anti-FcγRIIB + YFP rFVIIIFc, **(H)** 0.2 µg APC anti-FcγRIIB + YFP rFVIIIFc, **(I)** 0.1 µg APC anti-FcγRIIB + YFP rFVIIIFc, **(J)** 0.4 µg FVIII + YFP rFVIIIFc, **(K)** 0.2 µg FVIII + YFP rFVIIIFc, **(L)** 0.1 µg FVIII + YFP rFVIIIFc.
YFP = yellow fluorescent protein.

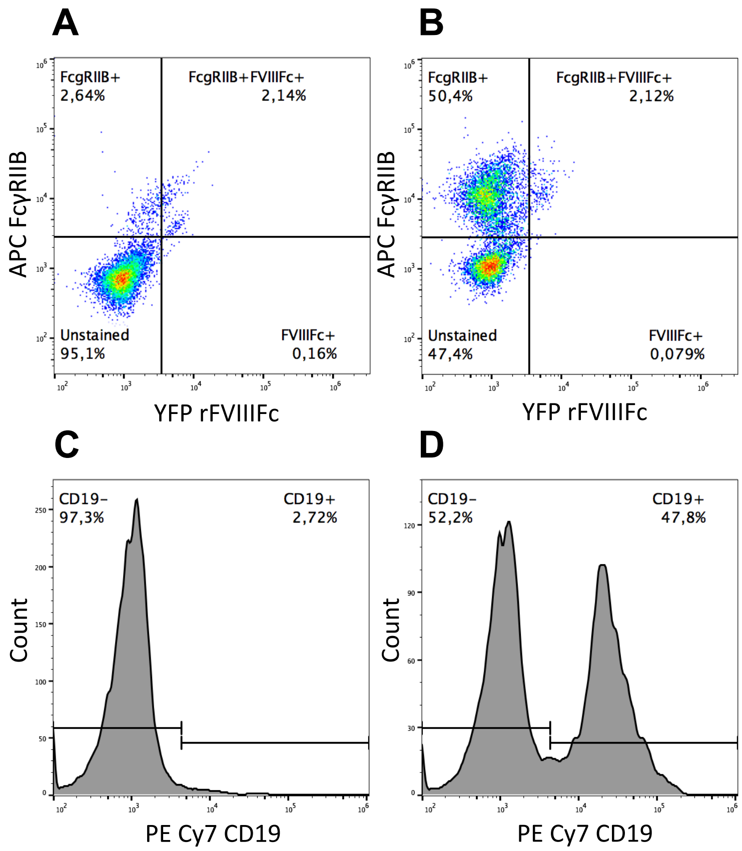

Supplementary Figure 3. Representative flow cytometry results of the rFVIIIFc binding assay isotype controls. FVIII-exposed lymphocytes stained with **(A)** APC mouse IgG2a isotype, **(B)** APC anti-FcγRIIB, **(C)** PE Cy7 Rat IgG2a isotype, **(D)** PE Cy7 anti-CD19.
